# The Role of Molecular Pathology in the Diagnosis of Cutaneous Lymphomas

**DOI:** 10.1155/2012/913523

**Published:** 2012-11-19

**Authors:** Philipp W. Raess, Adam Bagg

**Affiliations:** Department of Pathology and Laboratory Medicine, University of Pennsylvania, 7.103 Founders Pavilion, 3400 Spruce Street, Philadelphia, PA 19104-4283, USA

## Abstract

Primary cutaneous lymphomas can be difficult to be distinguished from reactive mimics, even when integrating histologic, immunophenotypic, and clinical findings. Molecular studies, especially PCR-based antigen receptor gene rearrangement (ARGR) analysis, are frequently useful ancillary studies in the evaluation of cutaneous lymphoproliferations. The biologic basis of ARGR studies is discussed, as well as a comparison of various current protocols. The pitfalls and limitations of ARGR analysis are also highlighted. Recent advances in the understanding of the molecular pathogenesis of various cutaneous lymphomas are discussed. Some of these nascent discoveries may lead to the development of diagnostically useful molecular assays.

## 1. Introduction

Cutaneous lymphoproliferations remain a challenging area to both dermatopathologists and hematopathologists despite concerted research efforts and recent diagnostic advances. The molecular pathogenesis of systemic lymphomas has been rigorously studied for some time, whereas cutaneous lymphomas were initially not given the same focus. However, over the last 5–10 years, cutaneous lymphomas have been the subject of intensive investigation at the genetic level [1]. Together with the standardization of diagnostic approaches and clinical classification, molecular analysis is likely to assume an increasing role in the evaluation of cutaneous lymphomas and their mimics. Indeed, molecular testing is already incorporated into recommendations for diagnosis and staging of cutaneous T-cell lymphoma (CTCL) [1–3]. The purpose of this paper is twofold: (1) to discuss the role and limitations of antigen receptor gene rearrangement studies and (2) to summarize recent developments in our understanding of the molecular pathogenesis of cutaneous lymphomas. 

## 2. Antigen Receptor Gene Rearrangements

### 2.1. Generation of Immunological Diversity

An understanding of the diagnostic utility of evaluating immunoglobulin and T-cell receptor gene (collectively referred to hereafter as antigen receptor genes (ARGs)) rearrangements is predicated upon an understanding of their normal biology. ARG structure and the processes through which they are rearranged are responsible for the ability of the adaptive immune system to identify a vast array of antigens. ARGs are composed of multiple variable (V), diversity (D), and joining (J) regions, followed by a constant (C) region. Several antigen receptor genes (*IGK@*, *IGL@*, *TRG@*, and *TRD@*) do not contain D regions. 

**Figure 1:**
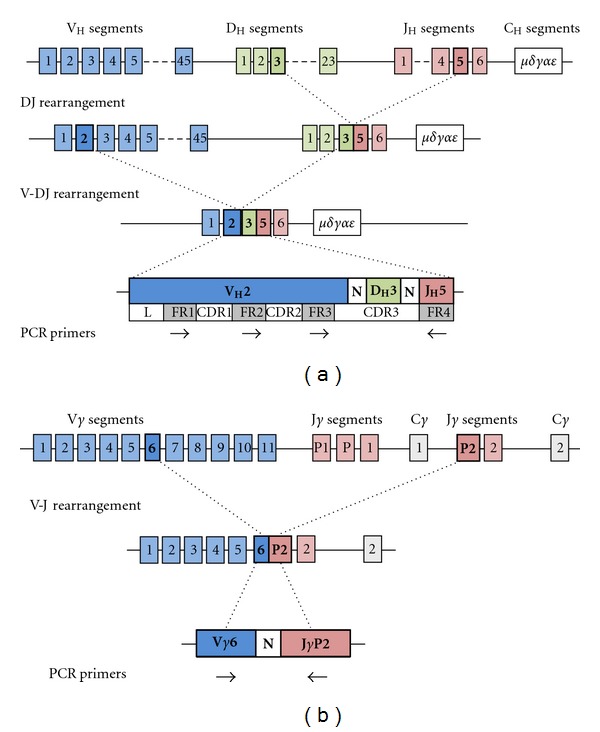
(a) *IGH@* gene structure, VDJ rearrangement, and PCR primer annealing sites. The *IGH@* gene is composed of ~45 V segments, ~23 D segments, and 6 J segments. DJ rearrangement occurs first, here combining D_H_3 and J_H_5. This is followed by V-DJ rearrangement, here combining V_H_2 with the previously rearranged D_H_3-J_H_5. Multiple PCR primers are directed against the 3 FR regions in the V segment and a single primer to the J segment (FR4). N represents nucleotides inserted and deleted by TdT. Adapted with permission from [4]. (b) *TRG@* gene structure, VJ rearrangement, and PCR primer annealing sites. The *TRG@* gene is composed of 11 V segments and 5 J segments. VJ rearrangement here combines the V*γ*6 and J*γ*P2 segments. Multiple PCR primers are directed against V*γ* segments and J*γ* segments.

In the *IGH@* gene, for example, the process of recombination begins with the alteration of tertiary gene structure to place a single D segment and a single J segment in close physical proximity, followed by the creation of a double-stranded DNA break and the rejoining of these D and J segments by recombination activating gene (RAG) proteins with the excision of intervening DNA (Figure 1). Terminal deoxynucleotidyl transferase (TdT) adds (and subtracts) several random nucleotides to the recombination site, thus increasing ARG diversity. Following D-J recombination, a similar process joins a V segment with the recombined D-J segment (followed by TdT action); D-J recombination always occurs before V-DJ recombination. The order of recombination of different ARGs is also typically hierarchical. In developing B cells, the *IGH@* gene undergoes recombination followed by the *IGK@* gene and, failing successful *IGK@* rearrangement, the *IGL@* gene. In developing T cells, the sequence of events is usually recombination at *TRD@*, then *TRG@*, *TRB@*, and finally *TRA@*. The sequence of these events has implications for detecting ARG rearrangements (ARGRs) in lymphoid neoplasms; the *IGH@* and *TRG@* loci are the most frequently evaluated in ARGR assays, since these are expected to be rearranged in the majority of B-cell and T-cell neoplasms, respectively. 

ARGR and the random insertion (and deletion) of nucleotides by TdT are largely responsible for generating the tremendous diversity required for a functional adaptive immune system. These two processes (recombinatorial and junctional diversity, respectively, at all seven different ARG loci) each leads to approximately 10^6^ different combinations, yielding the potential for as many as 10^12^ different ARGRs. The rearranged ARGs contain both framework regions (FRs), which correspond to structurally important portions of immunoglobulin protein and are conserved between genes, and complementarity determining regions (CDRs), which are variable between genes and largely determine antigen specificity. While two of the CDRs (1 and 2) are encoded in the germline of different V segments, CDR3 is generated by both recombinatorial and junctional diversity and represents the most heterogeneous region of the ARG and ultimately the immunoglobulin (Figure 1(a)) or T-cell receptor (Figure 1(b)) protein. The uniqueness of each rearranged ARG can be exploited as a molecular fingerprint used to determine if a lymphoproliferation is clonal or not. If all or many lymphocytes within a sample are an expansion from a single transformed lymphocyte (in other words, a lymphoid neoplasm), this population will share the same ARGR and is considered clonal. A polyclonal population, conversely, contains many lymphocytes, each with different ARGRs, as seen in reactive lymphoproliferations. Assays that determine the clonality of a lymphoid infiltrate examine the number of different ARGRs present, typically by differentiating ARGRs on the basis of size (or length). In the context of polymerase chain reaction (PCR) assays, these size differences are predominantly a consequence of the effects of TdT. 

### 2.2. Immunoglobulin and T-Cell Receptor Gene Rearrangement Analysis

Southern blot analysis of ARGR is no longer employed clinically due to laborious technical requirements including the use of radioactivity, high input DNA requirements (10–20 *μ*g), inability to be performed on formalin-fixed paraffin-embedded (FFPE) tissue (with the latter two issues especially constraining in skin biopsies), and relatively limited sensitivity (5–10%) [5, 6]. PCR-based techniques have supplanted Southern blot analysis [7, 8]; they require less input DNA, can be performed on FFPE tissue, and have a more rapid turnaround time. 

**Figure 2:**
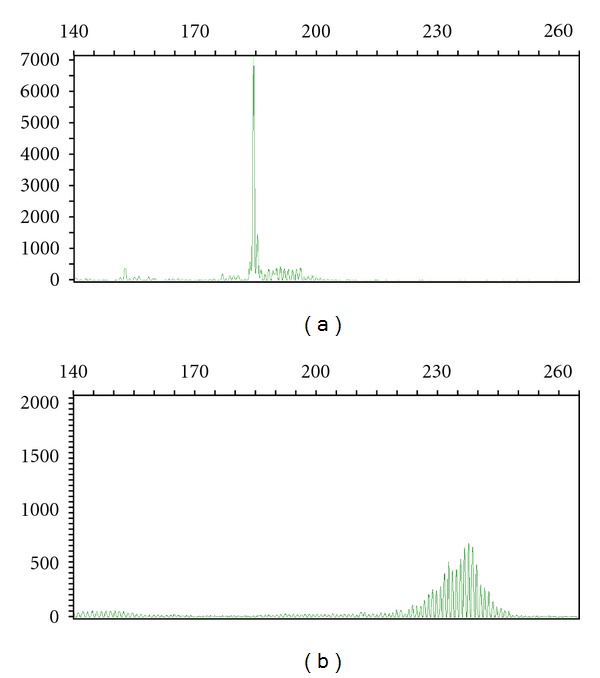
Capillary electropherograms of *TRG@* PCR studies. Clonal (a) and polyclonal (b) rearrangements (from different samples) are shown, using V*γ*9-11 primers and V*γ*1-8 primers, respectively. The *X* axis is amplicon size (base pairs) and the *Y* axis is random fluorescent units.

PCR-based strategies take advantage of the variability in ARGR size, which is primarily due to the variability in the size of CDR3 (in *IGH@*, e.g.) that in turn is largely the result of the actions of TdT (junctional diversity). PCR primers are directed against conserved FRs in the V and J regions; for CDR3 amplification, these are FR3 and FR4. Since long introns separate individual V regions from one another, only 5′ primers that anneal in the V region most proximal to the recombination site (namely, the one involved in the actual rearrangement) will create an amplicon that is sufficiently short to amplify efficiently. The same concept applies to 3′ primers and J regions. Amplicons are then separated based on their length in base pairs, typically via capillary electrophoresis. A sample is considered clonal if ARGs of only one size are detected (Figure 2(a)) and polyclonal if a Gaussian distribution of ARG sizes is detected (Figure 2(b)). Some cases may display ARGR patterns that are not straightforward to interpret; these are discussed in more detail later. Multiple algorithms have been proposed to determine whether a peak is significantly elevated above background [9, 10]. Some of these involve calculating the ratio of peak height to neighboring peaks or the expected Gaussian distribution. Uniformity has not been achieved in the application of methods for determining the presence of a “true” peak. Some groups, in particular the BIOMED consortium (see below), eschew the application of seemingly arbitrary numerical values to determine what makes a dominant peak monoclonal. We recommend a qualitative assessment of any peaks, in the context of a thorough understanding of assay limitations based on individual PCR protocols and both histologic and immunophenotypic features of the specimen, to determine whether a peak is meaningful.

Although simple in concept, the first generation of ARGR PCR assays initially posed several technical challenges since multiple differing approaches were described. The use of multiple target loci and genes, heterogeneous primer sets, different platforms for analysis of PCR products, and variation in sample types, amongst many other variables, made comparison between studies difficult. This technological heterogeneity led to challenges in determining optimal diagnostic approaches. In 2003, a multinational effort by the BIOMED consortium described standardized primer sets and methodology for the detection of clonal ARGRs [11]. This landmark study has served as a reference point for subsequent investigations into PCR-based assessment of clonality and as a guide for the development of clinical assays. The original description of the BIOMED-2 primer sets includes 107 primers in 18 multiplexed PCR reactions that interrogate both immunoglobulin (*IGH@* VH-JH, *IGH@* DH-JH, *IGK@*, and *IGL@*) and T-cell receptor gene rearrangements (*TRB@*, *TRG@*, and *TRD@*). Multiple updates in 2007 detailed assay refinements and performance in numerous neoplastic and reactive samples (summarized in [12]), albeit with a conspicuous paucity of cutaneous specimens. 

### 2.3. Performance of BIOMED-2 in Cutaneous Samples and Comparison with Other PCR-Based Assays

Following refinement of the BIOMED-2 protocol, the reported sensitivity of this assay was 99% for both B- and T-cell neoplasms [12]. The specificity of this protocol was reported to be 75% in a large set of histologically reactive lesions, with an additional 15% of samples representing “probably polyclonal” lesions [13]. However, the vast majority of specimens tested in these studies consisted of fresh/frozen nodal tissue. The performance of the BIOMED-2 protocol in more common FFPE samples and underrepresented tissues became a focus of future work. Multiple groups have investigated the performance of the BIOMED-2 protocol in cutaneous FFPE samples, with reported sensitivities ranging from 77% to 94% for T-cell neoplasms [14–16] and 85% for B-cell neoplasms [17]. The lower sensitivity of these studies is likely due to decreased DNA quality in FFPE tissue.

Although the BIOMED-2 protocol performs relatively well in FFPE cutaneous samples, adoption of this approach is far from universal. Multiple authors have proposed alternative PCR protocols and demonstrated their equivalency by direct comparison to the BIOMED-2 protocol in both fresh/frozen and FFPE cutaneous samples [14, 15, 18]. In a recent College of American Pathologists survey [19], approximately equal proportions of responding laboratories used the commercially available BIOMED-2 primer set versus other commercial or laboratory-developed assays for *IGH@* rearrangements. For *TRG@* analysis, a fewer laboratories (49 versus 66) used BIOMED-2 primer sets versus other assays, in particular laboratory-developed tests. The low rate of adoption of BIOMED-2 protocol may reflect its cost, increased complexity, or equivalent performance within certain sample types that are prevalent in an individual laboratory's case load.

ARGR studies, regardless of the protocol employed, are often of increased importance when the histologic and/or clinical appearance of a cutaneous lymphoid lesion has features that preclude clearly distinguishing a benign process from a lymphoma. In cutaneous specimens, analysis of T-cell receptor gene rearrangements (TCRGRs) is more commonly performed than that of immunoglobulin genes. This is partly because primary cutaneous T-cell neoplasms are more common than primary cutaneous B-cell neoplasms, but also because of the sometimes-overlapping histological features of CTCL and benign inflammatory dermatoses. In addition, T cells do not have an easily measurable immunophenotypic marker of clonality akin to immunoglobulin light-chain restriction in mature B-cell neoplasms. Finally, identifying a clonal ARGR is included in recommended algorithms for diagnosing mycosis fungoides (MF) [1, 3].

TCRGR testing has been most thoroughly investigated in differentiating MF from reactive inflammatory dermatoses. Early work demonstrated clonality by TCRGR in 76% of cases with initial suspicion of CTCL, but was limited by a small sample size (*n* = 29) [20]. Others have reported similar sensitivity (78%) and specificity (74%) in large series of CTCL and benign inflammatory dermatoses [21]. TCRGR testing has also been shown to be useful in differentiating specific subtypes of CTCL from their reactive histologic mimics. For example, granulomatous MF is included in the differential diagnosis of granuloma annulare and cutaneous sarcoidosis. In one series, clonal TCRGRs were identified in 13/14 (94%) cases of granulomatous MF and only 2/50 (4%) cases of granuloma annulare and sarcoidosis [16]. Concordantly, other authors have detected clonal TCRGR in only 4/29 (14%) of cutaneous granulomatous infiltrates [22]. 

ARGR testing, however, is not a panacea for histologically challenging cutaneous lymphoid infiltrates. Cutaneous lymphoid hyperplasia (CLH), also known as pseudolymphoma, is a brisk lymphoid infiltrate that is difficult to differentiate from a cutaneous lymphoma on clinical, histological, and immunophenotypic grounds [23]. Clonal ARGRs have been reported in 4%–61% of cases in several series of CLH; this wide range is likely due in part to differing histologic definitions of CLH, small sample sizes, and nonuniform PCR strategies [24–26]. Nonetheless, the preponderance of evidence indicates that ARGR testing is frequently a useful diagnostic test in the evaluation of cutaneous lymphoid infiltrates. 

### 2.4. Pitfall and Limitations of ARGR Studies

**Figure 3:**
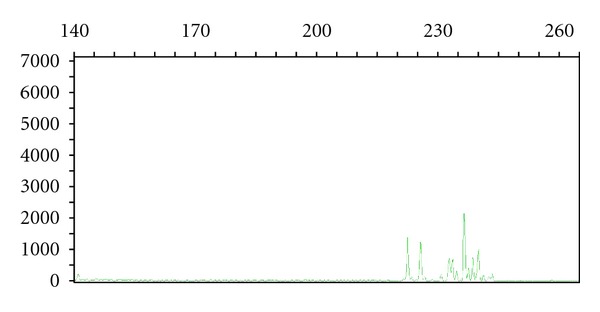
Capillary electropherogram of *TRG@* PCR study. Oligoclonal rearrangements using V*γ*1-8 primers. Note the presence of multiple irregular peaks in a nonGaussian distribution. The *X* axis is amplicon size (base pairs) and the *Y* axis is random fluorescent units.

PCR-based ARGR assays are susceptible to numerous biologic and technical factors that can complicate or confound analysis. In many instances, ARGR generates results that are either clearly clonal or polyclonal. However, ARGR assays may generate a pattern of peaks that is neither clonal nor polyclonal, a finding termed oligoclonality (Figure 3). Oligoclonal ARGR studies may represent the greatest interpretive challenges for both individual assay interpretation and integration of ARGR results into a meaningful diagnosis; importantly, this is not a failure of the assay *per se*, but rather a reflection of biology as a result of a limited antigen receptor repertoire found in some reactive processes.

Clonal heterogeneity within a lesion may also cause difficulty in interpreting ARGR studies. Conventional wisdom suggests that a maximum of two peaks (corresponding to biallelic rearrangement in a single clone) may be found in a single clonal process. However, clonal heterogeneity within a *bona fide* neoplasm can lead to increased numbers of peaks. This phenomenon is well documented in CTCL [27, 28].

False negative gene rearrangement PCR results are not uncommon and can result from various circumstances that result in poorly annealing PCR primers. Because of the large numbers of individual V-, D-, and J-segments in ARGs, it is not practical to include primers which are specific for every single segment. Rather, family-specific primers are used so that, for example, 6 or 7 *IGH@* V primers are used instead of ~45 individual V primers. However, several lymphomas have been demonstrated to have a nonrandom utilization of gene segments, so rational design of primer sets can optimize detection rates [29, 30]. 

Somatic hypermutation is a common cause of false negative IGH@ rearrangement studies found in B cells that originate from or have transited through the germinal center. As a naïve B cell interacts with antigen in the germinal center, point mutations are introduced by the enzyme activation-induced deoxycytidine deaminase (AID), primarily into CDR3. Should these mutations occur in an FR (typically FR3), PCR primers may not anneal effectively. This could result in an inefficient or ineffective PCR reaction and a false negative result. 

False positive gene rearrangement studies also pose challenges during diagnosis. If the ARG from an individual cell is preferentially amplified in a background that does not contain many other lymphoid cells (and hence limited ARGs), a “clonal” rearrangement may result. However, a repeat analysis will typically reveal the presence of a *differently sized* rearrangement; the inability to reproduce the detection of an identical monoclonal rearrangement is what defines pseudoclonality [31]. False positive results occur due to preferential PCR amplification of an individual amplicon within a limited pool of polyclonal ARGs. This concept was elegantly demonstrated by using laser capture microdissection of 10-10,000 lymphocytes from reactive and clonal processes [32]. Pseudoclonality was demonstrated when fewer than 2,000 lymphoid cells captured from a cutaneous biopsy of a reactive dermatitis were amplified via *TRG@ *PCR. In contrast, when more than 2,000 cells of the same lymphoproliferation were analyzed, *TRG@* PCR was polyclonal. Increased complexity of assay design (multiple tubes and/or fluorophores) has also been shown to decrease the specificity of TCRGR through a similar principle [33]. Division of a pool of ARGRs into multiple separately analyzed PCRs can result in a pseudoclonal spike being considered positive when compared to an artificially decreased polyclonal background. For these reasons, it is recommended that all PCR analyses are performed in duplicate and only those peaks that are reproducible in independent analyses can be considered to reflect definitive evidence of clonality. 

Clonal T-cell receptor rearrangements can be detected in B-cell neoplasms, and vice versa [34]. Clonal rearrangements of both T-cell receptor and immunoglobulin receptor genes have also been identified within the same neoplasm. These do not represent “true” false positives, but it is important to remember that ARGR should not be used to determine lineage.

### 2.5. Strategies and Recommendations to Improve Performance of PCR-Based Assays in Cutaneous Lymphoma

Despite its limitations, data gleaned from ARGR testing provide critical information in differentiating reactive from neoplastic lymphoproliferative cutaneous infiltrates. It is important to appreciate that such analyses are not perfect arbiters of neoplastic versus reactive lymphoproliferations, and that occasionally intermediate results actually reflect the inherent heterogeneity of the normal immune response. Nevertheless, a number of testing strategies have been proposed to increase the accuracy of ARGR PCR. 

Performing ARGR testing from multiple cutaneous sites and concurrent blood testing can be extremely useful, since the documentation of an identical clone in two or more sites increases the likelihood that it is indeed reflective of a *bona fide* neoplasm [28, 35, 36]. We recommend this strategy when multiple lesions are present and amenable to biopsy. Note that this should not substitute for performing the same PCR in duplicate on an individual site; this step remains critical in excluding pseudoclonality.

Not all cutaneous lymphoid infiltrates are ideal candidates for ARGR testing. Algorithms which tailor ARGR testing to the clinically determined pretest probability of CTCL have been advocated as a rational approach to this conundrum [21]. This approach provides the benefit of integrating clinical information with testing strategy and has been demonstrated to increase the positive and negative predictive value in a large series (*n* = 202 samples) of reactive and neoplastic lymphoid infiltrates. When the pretest probability of CTCL is moderately low, for example, a positive ARGR test increases the positive predictive value from 41% to 80%. The authors propose a diagnostic algorithm that maximizes sensitivity when pre-test probability is moderately low and maximizes specificity when pre-test probability is moderately high. Furthermore, the authors recommend against TCRGR testing in cases with very low or very high pre-test probability of MF. 

## 3. Molecular Pathogenesis of Cutaneous Lymphomas

The dissection of the molecular pathways involved in the development of cutaneous lymphomas has not proceeded at the same pace as it has in noncutaneous lymphomas. There may be a number of explanations for this, not the least of which is the typically small size of the lesions and challenges in accruing sufficient amounts of material to study. Nevertheless, a number of recent discoveries have been made that may aid the development of better diagnostic tools, refinement of prognostication, and targeted therapy. What follows is not intended to be a comprehensive review of all types of cutaneous lymphomas. The focus here is on those specific subtypes where molecular analysis might currently have practical applications.

## 4. T-Cell Neoplasms

### 4.1. Mycosis Fungoides

Several gene expression (mRNA) profiling studies have been performed in MF and have been correlated with patient outcomes. Additionally, mRNA expression studies also suggest potential therapeutic targets in MF lymphomagenesis. Early work in this arena implicated genes involved in the NF-*κ*B signaling pathway in the pathogenesis of MF, confirming *in vitro* studies [37, 38]. Multiple gene expression studies in MF followed; an interesting caveat of many of these studies is the heterogeneity of the tissue analyzed. Cutaneous biopsies rarely, if ever, contain a homogeneous population of neoplastic cells. The choice of control also markedly affects analysis; is normal skin the most appropriate control, or a reactive lymphoid infiltrate? In order to control for these variables, a meta-analysis of publicly available mRNA expression databases was performed from tumor-stage MF, isolated reactive T-cell subsets, normal skin, and skin with reactive inflammatory infiltrates [39]. Several hundred gene products were differentially expressed in tumor stage MF, including multiple gene products involved in the NF-*κ*B signaling pathway. Continued investigation of clues provided by gene expression data may potentially lead to a greater understanding of lymphomagenesis and rational development of targeted therapeutics. 

In addition to mRNA gene expression data, microRNA (miRNA) expression profiling has also identified numerous targets that are differentially expressed in MF versus reactive inflammatory dermatoses [40, 41]. A panel of five miRNAs identified through expression profiling has recently been shown to potentially differentiate reactive lymphoid infiltrates from neoplastic lesions in CTCL (primarily MF) [40]. While the majority of these studies have utilized array-based platforms, another approach is to use deep sequencing to analyze miRNA expression in peripheral blood lymphocytes in Sézary syndrome [42]. This may be advantageous in minimizing hybridization and other technical artifacts, but its utility in tissue-based samples with admixed reactive and neoplastic cells has yet to be demonstrated.

Comparative genomic hybridization, an array-based technology that identifies small chromosomal losses and gains at higher resolution than conventional cytogenetics, has also been applied to MF [43]. Multiple recurrent (>35% of samples) chromosomal abnormalities were identified (including gains on 7q, 7p, and 1q, losses on 5q, 9p, and 13q) that correlate well with mRNA expression data from these regions. Three chromosomal alterations were associated with decreased survival by univariate analysis in a relatively small data set of only 24 cases (9p21 loss, 8q24.3 gain, and 1q21-22 gain). A subsequent study used an approach with a much higher resolution and confirmed copy number alterations in samples of tumor-stage MF at some of the same loci as previously reported [44]. These alterations involve known protooncogenes and tumor suppressor genes (8q24.21 (*MYC*) gain, 9p21.3 (*CDKN2A*, *CDKN2B*, and *TMAP*) loss, and 10q26qter (*MGMT, EBF3*) loss) and also demonstrated prognostic differences. However, multivariate analysis did not demonstrate significant differences in prognosis. Whether these are primary or secondary genetic events is unknown. Nonetheless, the pathways involved may still be useful for the identification of efficacious targeted therapeutics. 

### 4.2. Primary Cutaneous Anaplastic Large Cell Lymphoma

Primary cutaneous anaplastic large cell lymphoma (PCALCL) has recently been the focus of several interesting, and potentially confusing, studies describing translocations involving 6p25, which are seen in 20–26% of cases [45, 46]. These translocations were first described in a series of peripheral T-cell lymphomas (PTCLs), including some PCALCLs [47], and initially the gene on 6p25 most commonly involved in PCALCL was presumed to be the nearby *IRF4* oncogene. However, subsequent analysis revealed that a slight majority of these 6p25.3 rearrangements in ALCL (both primary cutaneous and ALK-negative systemic) involve *DUSP22* [48], while fewer (~30%) do indeed involve *IRF4*. DUSP22 is a dual-specificity phosphatase that inhibits T-cell antigen receptor signaling and has been shown to have a tumor suppressor function in ALK-positive ALCL [49]. The translocation partner for *DUSP22* is the common fragile site *FRA7H* on 7q32.3 in almost one-half of these cases and remains unknown in the other described cases. 

Array CGH has also been performed in CALCL in numerous studies; however, no recurrent chromosomal gains or losses have been consistently identified across studies [50–53]. 

## 5. B-Cell Neoplasms

### 5.1. Primary Cutaneous Follicle Centre Lymphoma

Primary cutaneous follicle centre lymphoma (PCFCL) is a well-defined clinical entity that is surrounded by some controversy at the molecular level. Nodal follicular lymphoma, one of the most common lymphoma subtypes, has been thoroughly investigated and is characterized by the t(14;18)(q32;21) translocation involving the *IGH@* and *BCL2* genes in ~85-90% of cases. The prevalence of this translocation in PCFCL, however, has been the subject of much debate. Disparate results regarding the presence of t(14;18)(q32;21) have been reported and appear to be related, at least in part, to the analytic technique employed. Studies that have used PCR-based approaches have demonstrated the presence of the t(14;18)(q32;21) in 0 to 41% of cases, whereas studies using FISH report a prevalence of 0–51% [54–56]. Multiple variables complicate interpretation of these disparate results, including differing PCR strategies, potential inclusion of secondary cutaneous follicular lymphoma samples, and geographic variability. A direct comparison of PCR- and FISH-based approaches demonstrated the t(14;18)(q32;21) translocation in 11/27 cases by FISH and in 0/27 cases by PCR [56]. BCL2 expression by immunohistochemistry was noted in 10/27 cases (in cases both with the translocation and those without), in concordance with other published results [55]. The presence of BCL2 overexpression supports the role of the t(14;18)(q32;21) translocation in the pathogenesis and also implies other molecular mechanisms of BCL2 upregulation in translocation-negative cases, similar to what has been documented in nodal cases. Translocations involving the *IGH@* and *BCL6* genes (t(3;14)(q27;q32)) are also noted in a small fraction of PCFCL, again paralleling what is noted in nodal follicular lymphoma. Although better understanding of the pathogenesis of PCFCL will be gained through continued investigation into the underlying molecular biology, it is important to note that the prognosis of these patients is generally excellent whether or not a translocation is identified. 

### 5.2. Primary Cutaneous Diffuse Large B-Cell Lymphoma, Leg Type

When first described, primary cutaneous diffuse large B-cell lymphoma, leg type (PCDLBCL, LT) was differentiated from other primary cutaneous large B-cell lymphomas because of its later age of onset, frequent dissemination to noncutaneous sites, and worse prognosis, in addition to high frequency of primary lesions on the lower extremities. Further support for PCDLBCL, LT as a separate clinicopathologic entity was provided by CGH and gene expression data that demonstrated unique patterns distinct from other histologically similar PCBCLs [57–59]. The prevalence of chromosomal rearrangements involving 3q27 (*BCL6*), 8q24 (*MYC*), and 14q32 (*IGH@*) loci was found to be much higher in PCLDBCL, LT (11/14 cases) versus other PCBCL (0/15) [60]. Further work supported the concept that there are different patterns of chromosomal alterations between PCFCL, which can also display large cell morphology, and PCDLBCL, LT, identifying the loss of 9p21.3/*CDKN2A* through deletion in ~75% cases of PCDLBCL, LT [61]. This has been confirmed in other studies [62] and portends an adverse prognosis [63]. 

Point mutations in *MYD88* can be found in approximately two-thirds of PCDLBCL, LT cases, while none was identified in the cases of PCFCL and primary-cutaneous marginal zone lymphoma [64]. Although these findings emanate from a relatively small series that needs to be confirmed by larger studies, the specificity of this mutation in PCDLBCL, LT may provide a useful diagnostic molecular marker of this subtype of primary cutaneous B-cell lymphoma. However, *MYD88* mutations also occur in noncutaneous DLBCL, so it may be less useful in distinguishing PCDLBCL, LT from secondary cutaneous DLBCL [65]. 

### 5.3. Primary Cutaneous Marginal Zone Lymphoma

Genetically, primary cutaneous marginal zone lymphoma (PCMZL) differs from extranodal marginal zone lymphomas (mucosa associated lymphoid tissue/MALT lymphomas) arising in noncutaneous sites. The most frequent recurrent chromosomal abnormality in PCMZL is t(3;14)(p14.1;q32), although it occurs in only up to 10% of reported cases and is not specific to this site as it also occurs in ocular and thyroid extranodal MZL [66]. This translocation is thought to lead to the overexpression of *FOXP1* by *IGH@* enhancer elements. Other recurrent translocations in PCMZLs include t(14;18)(q32;q21) and t(11;18)(q21;q21), both affecting *MALT1*; however, these translocations are present in less than 15% of cases [60, 67, 68].

## 6. Conclusion

ARGR analysis is a useful assay to help characterize cutaneous lymphoproliferations. However, it is just one tool in the diagnostic armamentarium that we have at our disposal and needs, as always, to be integrated with the clinical picture, histology, and immunophenotype to make cogent diagnoses. It is not a perfect test and does not always provide results that definitively distinguish neoplastic from reactive lymphoid infiltrates. However, rather than viewing this as a failure of the assay (which, of course, is not flawless), it is important to appreciate that this may merely be reflective of the underlying biology and heterogeneity of the infiltrates themselves. Positive and negative predictive values are improved when these assays are used judiciously and appropriately. Finally, as the molecular pathways underlying specific subtypes of cutaneous lymphomas are dissected, we anticipate the development of novel assays that will be used in routine clinical practice to facilitate diagnosis and direct therapy.
